# Variability in mental health reporting among refugees and migrants in need of protection: new evidence from a weekly panel survey

**DOI:** 10.1186/s12889-023-15703-x

**Published:** 2023-05-05

**Authors:** Abigail Weitzman, Matthew Blanton, Gilbert Brenes Camacho

**Affiliations:** 1 Department of Sociology and Population Research Center, University of Texas at Austin, Austin, USA; 2 Centro Centroamericano de Población, University of Costa Rica, San José, Costa Rica

**Keywords:** Refugees, Migration, Mental health

## Abstract

**Background:**

The global population of refugees and other migrants in need of protection (MNP) is swiftly growing. Prior scholarship highlights that MNP have poorer mental health than other migrant and non-migrant populations. However, most scholarship on MNP mental health is cross-sectional, leaving open questions about temporal variability in their mental health.

**Methods:**

Leveraging novel weekly survey data from Latin American MNP in Costa Rica, we describe the prevalence, magnitude, and frequency of variability in eight indicators of self-reported mental health over 13-weeks; highlight which demographic characteristics, incorporation hardships, and violence exposures are most predictive of variability; and determine how variability corresponds to baseline mental health.

**Results:**

For all indicators, most respondents (> 80%) varied at least occasionally. Typically, respondents varied 31% to 44% of weeks; for all but one indicator they varied widely—by ~ 2 of 4 possible points. Age, education, and baseline perceived discrimination were most consistently predictive of variability. Hunger and homelessness in Costa Rica and violence exposures in origin also predicted variability of select indicators. Better baseline mental health was associated with less subsequent variability.

**Conclusions:**

Our findings highlight temporal variability in repeated self-reports of mental health among Latin American MNP and further highlight sociodemographic heterogeneity therein.

**Supplementary Information:**

The online version contains supplementary material available at 10.1186/s12889-023-15703-x.

## Background

More than 40 million people today are “migrants in need of protection” (MNP), whom the UNHCR defines as refugees, asylum-seekers, stateless persons, displaced Venezuelans or Syrians, and other internationally displaced “persons of concern” [1]. Because MNP typically migrate to evade imminent threats to their survival, many experience trauma before and sometimes during their migration [2]. Moreover, once abroad, many face protracted hardships such as hunger, housing instability, xenophobia, impermanent and/or uncertain immigration status, and family separation, among others [3].

Owing to these traumas and hardships, MNP exhibit disproportionate burdens of mental health issues (for reviews, see [4] and [5]). Nevertheless, most scholarship on MNP mental health is cross-sectional, leaving open questions about temporal *variability* in their mental health*.* Moreover, most studies of MNP are conducted among African, Arab, or Asian refugees in Europe, Australia, Canada, or the U.S. (for a review, see [6]). Existing scholarship thus largely overlooks Latin American refugees—a rapidly growing subpopulation [7]—and MNP who do not apply or qualify for refugee status or who live in lower- or middle-income destinations, where an estimated 83% resettle [8]. In light of these limitations, we collected and analyzed a weekly panel survey among MNP in Costa Rica—a Latin American, middle-income country where the number of MNP has grown 12-fold in the last five years [9].

## Methods

### Data

The *Encuesta de Refugiados: Experiencias Sociales y Salud* (ERESS; English translation: Survey of Refugees: Social Experiences and Health) consists of 260 adult MNP in Costa Rica.^1^ We recruited from October 2021 to April 2022 by disseminating information about the study at online workshops hosted by Fundación Mujer—one of the oldest NGO service providers of MNP in Costa Rica—and through snowballing. Interested individuals contacted the study team to provide a phone number where they could be surveyed and recontacted. The resultant sample is similar in mental health and incorporation experiences to a random sample of UNHCR-registered MNP in Costa Rica (Appendix A).

ERESS began with a 35-min baseline phone survey collecting information on respondents’ demographics; migration history; socioeconomic and legal incorporation; physical and mental health; and pre-migration violence exposures. After baseline, respondents were invited to complete 10-min weekly surveys online for twelve weeks. Survey questionnaires were informed by an extensive scope study that included more than a dozen interviews with MNP service providers and four focus groups and 34 in-depth interviews with MNP. Links to the followup survey were sent via Whatsapp using the phone number they provided. Participants received a $5 phone credit for the baseline survey and $5 phone credits each week they completed the online followup. 95% completed weekly followups; 78% completed followups through the last week. No significant differences in whether or how long someone participated in followups were detected across demographic characteristics or any independent variable in this study. Differential attrition was therefore unlikely to bias our conclusions. Given our emphasis on mental health *variability*, we focus on participants who completed at least one follow-up (*N* = 3,093 observations; 247 participants).

### Measures

*Mental health* was measured with eight questions. Each was derived from previously validated scales with one exception—global mental health^2^ —which was adapted from a previously validated question about global health more broadly. Potential responses ranged from (0) to (4) with higher numbers indicating greater intensity. Specifically, respondents were asked to rate their “current emotional or mental health;” “how satisfied [they were] with [their] life;” and how frequently, in the last week, they “easily felt nervous or scared.” Likewise, they were asked on a scale of (0) to (4) how much they agreed that they “have friends or family [they] can confide in;” “feel preoccupied, stressed, or angry about things that are out of [their] control,” “frequently feel down or sad;” “have trouble concentrating;” and “feel alone even when [they] are with others.”

For each, we generated three measures of variability: *any* captures whether participants ever provided dissimilar responses to the same question; *frequency* indicates the percentage of weeks participants varied from their previous answer; and *maximum* conveys the absolute difference between the highest and lowest score participants ever provided to a given question.

*Respondent demographics*, from baseline, include gender^3^; age; educational attainment; years since arrival; and number of dependents in Costa Rica.

*Incorporation* experiences, from baseline, include whether someone in the respondent’s household recently went hungry; if the respondent had been homeless in Costa Rica; if the respondent had ever had to do something that s/he “wasn’t proud of to survive or earn money;” and how often they felt discriminated against in the previous week, from (0) “never” to (4) “always.”

*Pre-migration violence exposure* sums the types of violence respondents ever experienced in origin (witnessed, extorted, persecuted, and threatened or harmed), ranging from 0 to 4.

### Analytic strategy

We first describe variability in mental health self-reporting during study participation. Following, we estimate bivariable models discerning how the likelihood, frequency, and magnitude of this variability differs with demographic characteristics, incorporation experiences, pre-migration violence exposure, and baseline mental health, where variability in each indicator is predicted by the baseline value of that same indicator.

## Results

At baseline, respondents reported moderate mental health, including averages of 3.01 in friends to confide in; 2.63 in stress; 2.45 and 2.41 in global mental health and life satisfaction, respectively; and 2.31 in sadness (Table 1). They reported low levels of nervousness (1.31) and were toward the middle for loneliness (2.09) and difficulty concentrating (1.97).

**Table 1:** Mental health means from baseline and weekly followups

	**Baseline** (*N* = 247)		**Followups** (*N* = 2,846)	
	Mean	95% CI	Mean	95% CI
Friends to confide in	3.01	(2.88 – 3.14)	2.86	(2.82 – 2.90)
Difficulty concentrating	1.97	(1.80 – 2.13)	1.96	(1.91 – 2.02)
Life satisfaction	2.41	(2.27 – 2.56)	2.63	(2.59 – 2.67)
Loneliness	2.09	(1.92 – 2.25)	1.79	(1.73 – 1.84)
Sadness	2.31	(2.16 – 2.46)	2.08	(2.04 – 2.13)
Stress	2.63	(2.49 – 2.78)	2.42	(2.37 – 2.47)
Nervousness	1.31	(1.15 – 1.47)	1.25	(1.20 – 1.30)
Mental health (global)	2.45	(2.34 – 2.56)	2.64	(2.60 – 2.67)

**Fig. 1:**
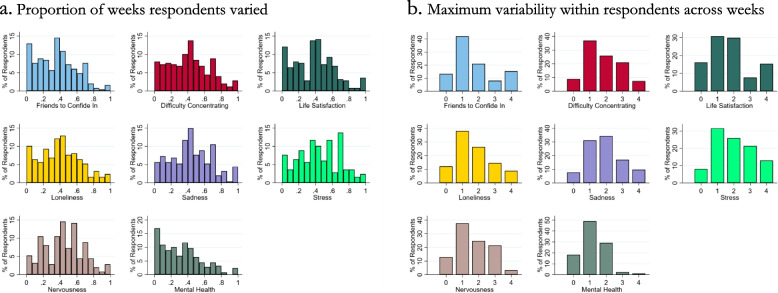
Variation in Mental Health during ERESS. **a**. Proportion of weeks respondents varied, **b**. Maximum variability within respondents across weeks

Respondents’ mental health during weekly followups appeared similar to baseline (Table 1). However, 87% of respondents varied at least once in their perceptions of having friends to confide in (Fig. 1a) and nearly 25% varied upwards of 3 or 4 points (Fig. 1b). Similar or greater percentages ever varied in self-reported difficulty concentrating, life satisfaction, loneliness, and global mental health (Fig. 1a). Likewise, a similar percentage varied maximally by 3 or 4 points with respect to all mental health indicators except global mental health (Fig. 1b). Thus, across numerous indicators, self-reported mental health varied at least occasionally; and for some, it varied widely.

As Table 2 conveys, > 90% of respondents ever varied in their self-reports of how much difficulty they had concentrating, felt sad, and felt stressed. For all other indicators, > 80% of respondents ever varied. Respondents varied most frequently—44% of times they were asked—about their sadness, stress, and nervousness. They similarly varied in 41% and 39% of times they were asked about difficulties concentrating and loneliness, respectively. They varied least often—31% of times—in global mental health (Table 2). Analogous patterns emerged in terms of maximum variation between respondents’ lowest and highest scores (Table 2). Thus, respondents were not just more likely to vary in some mental health indicators than in others, but also varied more frequently and widely in those same indicators.

**Table 2:** Descriptive Statistics of ERESS Respondents (*N* = 247)

	Mean/ Perc	95% CI
**Mental Health: Any variation (yes/no)**		
Friends to confide in	87	-
Difficulty concentrating	91	-
Life satisfaction	84	-
Loneliness	88	-
Sadness	92	-
Stress	92	-
Nervousness	87	-
Mental Health	82	-
**Mental Health: Frequency of variability (% of weeks)**		
Friends to confide in	35	(32—38)
Difficulty concentrating	41	(38—44)
Life satisfaction	38	(35—41)
Loneliness	39	(36—42)
Sadness	44	(40—47)
Stress	44	(41—47)
Nervousness	44	(40—46)
Mental Health	31	(28—34)
**Mental Health: Maximum variability across weeks (0–4)**		
Friends to confide in	1.70	(1.54—1.86)
Difficulty concentrating	1.81	(1.68—1.95)
Life satisfaction	1.75	(1.59—1.91)
Loneliness	1.70	(1.56—1.84)
Sadness	1.90	(1.76—2.03)
Stress	2.00	(1.85—2.14)
Nervousness	1.64	(1.51—1.77)
Mental Health	1.19	(1.09—1.29)
**Demographics**		
Gender		
Woman	66	-
Man	34	-
Age		
< = 24	11	-
25–34	25	-
35–44	36	-
45–54	16	-
> = 55	12	-
Educational		
< high school	17	-
High school	31	-
> = college	52	-
Years since Arrival in CR	3.44	(3.03 – 3.84)
# of dependents in CR	1.47	(1.32 – 1.63)
**Incorporation hardships**		
Someone in household recently went hungry	41	-
Ever homelessness since arrival	48	-
Had to do something not proud of since arrival	49	-
Frequency of discrimination in last week (0–4)	.64	(.51 – .77)
**Pre-migration violence exposure scale** (0–4)	2.08	(1.94 – 2.24)

Table 3 presents results of bivariable analyses examining predictors of mental health variability. Looking within columns reveals which baseline characteristics were predictive of volatility in a given indicator. The first column, for example, indicates that perceived friends to confide in varied less among older than younger respondents. It also varied less in magnitude and frequency among those who were college educated (versus not having completed high school) and with higher violence exposure in origin. Compared to those who had been in Costa Rica for < 1 year, respondents who were in-country for 1 to < 2 years were more likely to ever vary in perceived friends to confide in, as were those living in households where someone had recently gone hungry. Respondents living in households marked by hunger, who had been homeless in Costa Rica, had done something they weren’t proud of to survive, or felt more discriminated against varied more frequently in their perceptions of having friends to confide in than other respondents.

**Table 3:** Results of bivariable regressions predicting variability in mental health

	Friends to confide in			Difficulty concentrating			Life satisfaction			Loneliness			Sadness			Stress			Nervousness			Global mental health		
	Any	Size	Freq	Any	Size	Freq	Any	Size	Freq	Any	Size	Freq	Any	Size	Freq	Any	Size	Freq	Any	Size	Freq	Any	Size	Freq
*Coefficients*																								
**Demographics**																								
Woman	.03	.13	.01	.04	.03	-.02	.04	.19	-.03	-.01	-.09	.05	.04	.08	.03	-.04	-.15	.00	**.11**	**.31**	.06	.06	.03	.01
Age (ref: 18–24)																								
25–34	-.13	-.22	**-.11**	-.07	-.32	-.10	.01	-.38	-.04	-.13	-.31	-.09	.00	.05	-.07	.00	-.10	-.08	-.09	-.24	-.10	-.12	-.25	-.09
35–44	**-.17**	**-.57**	**-.18**	-.05	-.16	-.06	.00	-.24	-.08	-.10	-.16	-.08	.03	.22	-.03	.00	.00	-.04	-.05	-.42	-.10	**-.18**	-.32	-.10
45–54	-.08	-.49	**-.18**	-.01	-.12	-.06	.00	-.47	-.09	-.15	-.41	-.11	.00	-.29	**-.13**	.00	-.04	-.09	-.05	-.29	-.08	-.12	-.35	-.09
> = 55	**-.24**	**-.98**	**-.28**	-.10	**-.59**	-.12	-.09	**-.87**	-.12	**-.24**	**-.83**	**-.20**	-.10	-.26	**-.16**	**-.03**	-.07	-.09	-.03	-.48	-.11	**-.27**	**-.60**	**-.14**
# dependents in CR	-.01	.03	.01	.00	.06	.01	.04	.03	.02	.02	.05	.02	.03	**.14**	.02	-.01	-.06	-.02	.01	-.01	.00	.01	.04	.01
Years since arrival (ref: < 1)																								
1- < 2	**.26**	.46	.08	.16	**.80**	.05	.15	.70	.06	.04	.41	.03	.10	**.97**	-.03	.11	.60	-.04	.21	.63	.08	-.07	.05	-.03
> = 2	.13	.47	.03	.07	.33	-.04	.11	.14	.00	.10	.36	.02	.09	.42	-.01	.02	.25	-.05	.14	.39	-.01	.10	.29	.05
Education (ref: < H.S.)																								
Completed HS	-.04	-.20	.00	.07	-.01	.02	-.01	-.48	-.07	.01	-.42	-.03	.04	-.10	-.01	-.05	-.29	-.07	-.02	-.05	.01	-.07	.18	-.06
College or more	-.10	**-.46**	**-.10**	-.03	**-.63**	**-.09**	-.04	**-.60**	-.10	.03	**-.64**	**-.12**	.01	**-.45**	**-.09**	-.04	**-.42**	-.07	-.02	-.21	-.06	-.09	**-.33**	**-.13**
**Incorp. hardships**																								
Hunger	**.10**	**.45**	**.11**	**.08**	**.34**	**.07**	**.13**	**.32**	**.07**	.01	.18	.05	.04	.02	.05	.04	-.14	.03	.02	.15	.00	.08	.19	**.09**
Homeless	.08	.19	**.06**	**.08**	**.43**	**.09**	**.10**	**.33**	**.10**	.03	**.36**	.04	.02	.15	.04	.04	.23	.06	.03	.24	.04	.08	.16	**.04**
Not proud	.06	.32	**.12**	.02	.00	.03	**.16**	.05	**.13**	-.05	-.20	-.02	-.01	-.03	.02	-.03	-.22	.00	.01	.19	.07	.01	.07	.05
Discrimination	.02	.15	**.03**	**.03**	**.16**	.02	.00	.00	.00	.01	**.20**	.01	.01	**.19**	.02	.02	**.20**	.02	**.04**	**.16**	**.03**	.03	**.12**	.02
**Pre-mig. vio. scale**	-.03	**-.17**	**-.03**	-.03	-.09	.00	-.01	-.05	.01	**-.04**	-.12	**-.04**	-.02	**-.17**	-.01	**-.04**	**-.15**	**-.03**	-.03	-.09	-.01	-.01	-.02	-.01
**Base. MH indic**	-.04	.-.11	**-.05**	.01	**.11**	**.01**	**-.07**	-.12	-.03	**.03**	**.13**	.02	.00	.05	.01	.01	-.10	.00	**.06**	**.26**	**.05**	**-.10**	**-.24**	**-.05**
*95% CIs*																								
**Demographics**																								
Woman	(-.06- .12)	(-.20- .46)	(-.06- .07)	(-.03- .12)	(-.26- .32)	(-.09- .04)	(-.06- .13)	(-.14- .52)	(-.10- .03)	(-0.1- .08)	(-.38- .21)	(-.02- .11)	(-.03- .11)	(-.21- .36)	(-.03- .10)	(-.11- .04)	(-.46- .16)	(-.07- .07)	(.02- .19)	(.03- .58)	(.00- .12)	(-.04- .16)	(-.18- .24)	(-.05- .08)
Age (ref: 18–24)																								
25–34	(-.28- .03)	(.78- .35)	(-.21- .00)	(-.20- .06)	(-.82- .18)	(-.21- .01)	(-.16- .18)	(-.95- .19)	(-.15—.08)	(-.27- .02)	(-.83- .20)	(-.20—.02)	(-.12- .12)	(-.44- .54)	(-.18- .05)	(-.13—.12)	(-.64- .44)	(-.20—.03)	(-.24- .06)	(-.73-.24)	(-.20- .01)	(-.30- .06)	(-.61- .11)	(-.21- .02)
35–44	(-.32- -.02)	(-1.1- -.03)	(-.28- -.01)	(-.18- .07)	(-.64- .32)	(-.17- .05)	(-.17- .16)	(-.79- .31)	(-.19- .03)	(-.24- .04)	(-.65- .33)	(-.18- .03)	(-.09- .15)	(-.25- .69)	(-.13- .08)	(-.12- .12)	(-.52- .51)	(-.15- .07)	(-.20- .10)	(-.89- .04)	(-.2—.00)	(-.34- -.01)	(-.67—.03)	(-.20—.01)
45–54	(-.25—.09)	(-1.1—.13)	(-.29- -.07)	(-.16—.13)	(-.66—.42)	(-.19—.06)	(-.19—.19)	(-1.1—.15)	(-.22—.03)	(-.32—.01)	(-.97—.15)	(-.23—.01)	(-.13—.13)	(-.83—.24)	(-.25- -.01)	(-.14—.14)	(-.63—.55)	(-.21—.04)	(-.22—.12)	(-.82—.23)	(-.20- .03)	(-.31- .08)	(-.74- .05)	(-.21- .04)
> = 55	(-.42- -.06)	(-1.6- .33)	(-.40- -.16)	(-.25- .05)	(-1.2- -.01)	(-.25- .02)	(-.28- .11)	(-1.5- -.21	(-.25- .01)	(-.41- -.01)	(-1.4- -.23)	(-.33- -.07)	(-.24- .05)	(-.83- .30)	(-.29- -.03)	(-.17- .12)	(-.70- .57)	(-.22- .05)	(-.21- .15)	(-1.0- .08)	(-.24- .01)	(-.48- -.07)	(-1.0- -.18)	(-.27- -.01)
# dependents in CR	(-.06- .03)	(-.12- .18)	(-.02- .04)	(-.03- .04)	(-.07- .20)	(-.02- .04)	(-.01- .08)	(-.12- .19)	(-.01- .05)	(-.02- .06)	(-.08- .19)	(-.01- .05)	(-.01- .06)	(.01- .27)	(-.01- .05)	(-.04- .02)	(-.21- .08)	(-.05- .01)	(-.03- .05)	(-.14- .12)	(-.03- .02)	(-.04- .06)	(-.05- .14)	(-.02- .04)
Years since arrival (ref: < 1)																								
1- < 2	(.04- .48)	(-.35- 1.3)	(-.07- .24)	(-.03- .34)	(.10- 1.5)	(-.11- .21)	(-.09- .39)	(-.12- 1.5)	(-.10- .22)	(-.17- .26)	(-.33- 1.1)	(-.13- .19)	(-.07- .28)	(.28- 1.7)	(-.20- .13)	(-.07- .28)	(-.16- 1.4)	(-.20- .12)	(-.01- .42)	(-.06- 1.3)	(-.07- .23)	(-.32- .18)	(-.47- .57)	(-.19- .13)
> = 2	(-.03- .29)	(-.12- 1.1)	(-.08- .14)	(-.07- .20)	(-.19- .84)	(-.15- .08)	(-.07- .28)	(-.45- .74)	(-.12- .12)	(-.05- .26)	(-.18- .89)	(-.10- .14)	(-.04- .21)	(-.09- .92)	(-.12- .11)	(-.11- .15)	(-.30- .81)	(-.17- .07)	(-.02- .30)	(-.1- .90)	(-.12- .10)	(-.09- .28)	(-.10- .67)	(-.07- .16)
Education (ref: < H.S.)																								
Completed HS	(-.16- .09)	(-.67—.27)	(-.90—.09)	(-.04—.17)	(-.40—.39)	(-.07—.11)	(-.15—.13)	(-.95—.01)	(-.16—.02)	(-.12—.13)	(-.84—.00)	(-.13—.06)	(-.06—.14)	(-.50—.31)	(-.10—.09)	(-.15—.06)	(-.73—.15)	(-.16—.03)	(-.14—.11)	(-.45—.35)	(-.08—.10)	(-.22—.08)	(-.48—.12)	(-.15—.03)
College or more	(-.22—.02)	(-.90- .03)	(-.19- -.02)	(-.13- .07)	(-1.0- -.26)	(-.17- .00)	(-.17- .09)	(-1.0- -.16)	(-.18- -.01)	(-.08- .14)	(-.10- -.26)	(-.20- -.03)	(-.09- .10)	(-.82- -.08)	(-.18- -.01)	(-.13- .06)	(-.82- -.01)	(-.16- .01)	(-.13- .10)	(-.58- .16)	(-.14- .02)	(-.22- .05)	(-.60- -.05)	(-.22- -.05)
**Incorp. hardships**																								
Hunger	(.01- .18)	(.14- .76)	(.05- .17)	(.00- .15)	(.06- .61)	(.01- .13)	(.04- .22)	(.00- .63)	(.01- .14)	(-.07- .09)	(-.11- .46)	(-.01- .11)	(-.03- .10)	(-.26- .29)	(-.01- .11)	(-.03- .11)	(-.43- .15)	(-.03- .10)	(-.06- .11)	(-.12- .41)	(-.06- .05)	(-.02- .17)	(-.01- .39)	(.03- .15)
Homeless	(.00- .17)	(-.12- .51)	(.00- .12)	(.00- .15)	(.17- .70)	(.03- .15)	(.01- .20)	(.02- .65)	(.04- .16)	(-.06- .10)	(.07- .64)	(-.02- .11)	(-.05- .09)	(-.12- .42)	(-.02- .11)	(-.02- .11)	(-.06-.53)	(-.01- .12)	(-.06- .11)	(-.02- .50)	(-.01—.10)	(-.02- .18)	(-.04—.36)	(.03—.15)
Not Proud	(-.04- .16)	(-.05- .70)	(.05- .18)	(-.06 .10)	(-.33- .32)	(-.05- .10)	(.06- .27)	(-.33- .42)	(.06- .20)	(-.14-.05)	(-.54- .13)	(-.09- .06)	(-.09- .07)	(-.35- .30)	(-.05- .10)	(-.11- .05)	(-.57- .13)	(-.08- .07)	(-.09- .11)	(-.12- .50)	(.00- .13)	(-.10—.12)	(-.17- .31)	(-.02- .13)
Discrimination	(.02- .06)	(.00- .30)	(.00- .06)	(-00- .07)	(.03- .30)	(-.01- .04)	(-.05- .04)	(-.15- .15)	(-.03- .03)	(-.03- .05)	(.07- .33)	(-.02- .04)	(-.02- .04)	(.06- .32)	(-.01- .05)	(-.01- .05)	(.06- .33)	(-.01- .05)	(.00- .08)	(.03- .29)	(.00- .06)	(-.02- .07)	(.02- .21)	(-.01- .05)
**Pre-mig. vio. scale**	(-.06- .01)	(-.31- -.04)	(-.06- .01)	(-.06- .00)	(-.21- .03)	(-.03- .02)	(-.05- .03)	(-.18- .09)	(-.01- .04)	(-.07- .00)	(-.24- .00)	(-.07- -.01)	(-.05- .01)	(-.29- -.06)	(-.04 -.02)	(-.07- -.01)	(-.28- -.03)	(-.06- -.01)	(-.07- .00)	(-.20- .02)	(-.30- .02)	(-.06- .03)	(-.10- .07)	(-.03- .02)
**Base. MH indic**	(-.08- .00)	(-.26- .04)	(-.07- -.02)	(-.02- .03)	(.01- .22)	(-.02- .03)	(-.11- -.03)	(-.26- .02)	(-.05- .00)	(.00- .06)	(.02- .24)	(-.01- .04)	(-.03- .03)	(-.06- .16)	(-.01- .04)	(-.03- .04)	(-.23- .03)	(-.03- .03)	(.03- .09)	(.16- .36)	(.03- .07)	(-.15- -.04)	(-.35- -.13)	(-.09- -.02)

For all models, *N* = 247. Baseline MH indicator refers to the baseline value of the mental health indicator in a given column. (For example, in the first column, this indicator captures baseline friends to confide in. In the second column, it captures baseline difficulty concentrating, and so on.) (*p* < .05, two-tailed tests of significance)

For the most part, variability in self-reported difficulties concentrating, loneliness, and sadness shared similar predictors (with several exceptions, Table 3). Variability in self-reported stress, nervousness, and global mental health, on the other hand, corresponded with the fewest baseline indicators. Variability in all three was nonetheless associated with respondents’ education and perceived discrimination in Costa Rica.

Looking across Table 3 horizontally elucidates which of respondents’ characteristics were, on the whole, most predictive of temporal variation in self-reported mental health. For example, being among the oldest respondents was negatively associated with variability in all indicators except nervousness. Relative to respondents who had not completed high school, those who had graduated college were more stable in all indicators except nervousness. Conversely, the more discriminated against a respondent felt at baseline, the more s/he waivered in all indicators except life satisfaction. Hunger and past homelessness were both associated with more variability in perceived friends to confide in, difficulty concentrating, life satisfaction, and global mental health. Finally, though differing from one indicator to the next, the overall pattern of coefficients on baseline mental health suggests greater temporal variability among individuals reporting generally poorer mental health at baseline.

## Discussion

MNP commonly endure protracted traumas, adversities, and uncertainties, yet much remains unknown about their mental health variability. Adult mental health has notable implications for suicidality [10], somatization [11, 12], longer-term physiological wellbeing [13], and the health and wellbeing of child dependents [14]. Here, we offered one of the first assessments of weekly variability in adult MNP’s self-reported mental health.

Participants were most likely to ever vary—and varied greatest in magnitude and frequency—in difficulties concentrating, sadness, and stress. Nevertheless, temporal variation was highly common (> 80%) for *all* mental health indicators; and, on average, respondents varied between 1.6 and 2.0 out of 4 possible points between their highest and lowest self-rating for each. Thus, MNP typically exhibited regular and wide variation across most mental health indicators,suggesting that cross-sectional studies miss substantial temporal variation. Reassuringly, however, this variability is not dramatic enough to yield substantially different estimates when MNP mental health is averaged across repeated observations versus assessed only once (at baseline). Our findings thusly suggest that cross-sectional estimates of MNP mental health are likely reliable despite their cross-sectional nature.

Nonetheless, age, education, and perceived discrimination were consistently predictive of variability in numerous mental health indicators. Hunger and homelessness, as well as pre-migration violence exposures and baseline mental health were also predictive of variability of select indicators. These findings draw attention to the fact that MNP mental health variability relates to both their contemporary stressors and pre-migration traumas. Meanwhile, age and education-related heterogeneity highlights potential stratification in MNP mental health *trajectories* and *instability,* raising questions about how human capital, pre-migration trauma, and post-migration hardship jointly affect changes in MNP mental health over time and how these processes differ across the life course. Furthermore, they suggest that practitioners may eventually be able to screen for and project less stable mental health trajectories based on the demographic traits and pre-migration experiences of individual MNP.

This study, however, faces several limitations. First, our convenience sample is unlikely population representative of MNP in Costa Rica. Second, although we rely on previously validated measures to assess multiple dimensions of mental health, we rely on just one rather than a multitude of indicators to assess each dimension. It is therefore possible that when these dimensions are more comprehensively assessed, they exhibit more or less variability than we document here. Third, because our indicators are self-reported, they are subject to cultural interpretation and differences therein. For instance, the only gender difference in variability we observe is with respect to nervousness. One reason for differences in nervousness variability but no other indicators may have to do with gender norms that discourage men more than women from feeling or exhibiting fear. Fourth, our sample is limited to migrants. This prevents us from comparing variability in the self-reported mental health of MNP and non-migrants.

## Conclusion

Our findings underscore the need for research exploring the dynamic predictors and implications of MNP mental health variability for immediate and longer-term physical somatization, physiological wellbeing, interpersonal relationships, and resettlement intentions. For both researchers and clinicians, our findings indicate that MNP who are young adults, who have not completed high school, who feel more frequently discriminated against, and who have poorer baseline mental health exhibit higher variability in their self-reported mental health. Moreover, they suggest that MNP are most likely to ever vary and vary most widely and frequently in their difficulties concentrating, sadness, and stress levels.

## Supplementary Information

**Additional file 1. **

## Data Availability

The datasets generated during and/or analyzed during the current study are not publicly available due to the sensitive nature of this study’s content, ethical concerns, and the study’s IRB protocol, which restricts data sharing except among the study team and collaborators. Questions about the data, including to request access, should be directed to Dr. Abigail Weitzman at aweitzman@utexas.edu.

## Abbreviations

UNHCR: United Nations High Commissioner for Refugees
MNP: Migrants in need of international protection

## Footnotes

1 To participate, migrants had to be >  = 18 years old. > 97% had applied for asylum; reported that danger, insecurity, or political turmoil in their country was a key reason they migrated; and/or migrated from a country with a large humanitarian crisis in the year they migrated. Restricting the sample to these participants only yields nearly identical results.

2 These include: Satisfaction with Life Scale, Post Traumatic Symptom Scale Self-Report, UCLA Loneliness Scale, Perceived Stress Scale, Center for Epidemiologic Studies-Depression Scale, Impact of Event Scale, and Social and Emotional Loneliness Scale.

3 Three individuals identified as trans-women.
