# Supplementary material for: Variability in mental health reporting among refugees and migrants in need of protection: new evidence from a weekly panel survey
Source: BMC Public Health. 2023 May 5;23:832. doi: 10.1186/s12889-023-15703-x (PMC10161428; doi:10.1186/s12889-023-15703-x)
Supplement: Supplementary file 1 — Additional file 1. [file 12889_2023_15703_MOESM1_ESM.docx]

**Appendix A.** Comparison of Means in ERESS to UNHCR High Frequency Surveys

|  | **ERESS**  (N=260) |  | **UNHCR**  (N=9,271) |
| --- | --- | --- | --- |
| *Demographics* |  |  |  |
| Age |  |  |  |
| <=21 | .06 |  | .07 |
| 22-59 | .89 |  | .89 |
| >=60 | .06 |  | .04 |
| Any child dependents in Costa Rica | .50 |  | .52 |
| Nationality |  |  |  |
| Other | .16 |  | .15 |
| Nicaraguan | .50 |  | .76 |
| Venezuelan | .34 |  | .09 |
| Gender |  |  |  |
| Man | .33 |  | .48 |
| Woman | .67 |  | .52 |
| Relationship status |  |  |  |
| Married/ cohabiting | .55 |  | .34 |
| Other | .11 |  | .04 |
| Single | .34 |  | .62 |
| *Migration status and history* |  |  |  |
| Immigration status |  |  |  |
| Undocumented | .04 |  | .02 |
| Other visa | .14 |  | .05 |
| Humanitarian | .82 |  | .93 |
| Year of arrival |  |  |  |
| <=2017 | .21 |  | .16 |
| 2018 | .39 |  | .28 |
| 2019 | .23 |  | .24 |
| 2020 | .03 |  | .09 |
| >=2021 | .14 |  | .23 |
| *Hardships and Incorporation in Costa Rica* |  |  |  |
| Hungry | .48 |  | .51 |
| Kids enrolled in school | .80 |  | .89 |
| Receiving benefits from religious group | .02 |  | .02 |
| Loneliness (1 to 5) | 3.10 |  | 3.03 |

*Note:* Loneliness has been rescaled from 1 to 5 (instead of 0 to 4) in order to match the range in the UNHCR survey. UNHCR data come from three pooled standardized surveys conducted in 2022. For more information, visit https://microdata.unhcr.org/index.php/catalog/655.
